# Is using multiple imputation better than complete case analysis for estimating a prevalence (risk) difference in randomized controlled trials when binary outcome observations are missing?

**DOI:** 10.1186/s13063-016-1473-3

**Published:** 2016-07-22

**Authors:** Mavuto Mukaka, Sarah A. White, Dianne J. Terlouw, Victor Mwapasa, Linda Kalilani-Phiri, E. Brian Faragher

**Affiliations:** Malawi-Liverpool-Wellcome Trust Clinical Research Programme, College of Medicine, University of Malawi, Box 30096, Blantyre 3, Malawi; Department of Public Health, College of Medicine, University of Malawi, P/Bag 360, Blantyre 3, Malawi; Liverpool School of Tropical Medicine, Pembroke Place, L3 5QA Liverpool, UK; Mahidol-Oxford Tropical Medicine Research Unit, Mahidol University, 60th Anniversary Chalermprakiat Building, 3rd Floor, 420/6 Ratchawithi Rd, Bangkok, 10400 Thailand

**Keywords:** Missing binary outcome, Risk difference, Complete case analysis, Multiple imputation, Missing completely at random, Missing at random, Missing not at random

## Abstract

**Background:**

Missing outcomes can seriously impair the ability to make correct inferences from randomized controlled trials (RCTs). Complete case (CC) analysis is commonly used, but it reduces sample size and is perceived to lead to reduced statistical efficiency of estimates while increasing the potential for bias. As multiple imputation (MI) methods preserve sample size, they are generally viewed as the preferred analytical approach.

We examined this assumption, comparing the performance of CC and MI methods to determine risk difference (RD) estimates in the presence of missing binary outcomes. We conducted simulation studies of 5000 simulated data sets with 50 imputations of RCTs with one primary follow-up endpoint at different underlying levels of RD (3–25 %) and missing outcomes (5–30 %).

**Results:**

For missing at random (MAR) or missing completely at random (MCAR) outcomes, CC method estimates generally remained unbiased and achieved precision similar to or better than MI methods, and high statistical coverage. Missing not at random (MNAR) scenarios yielded invalid inferences with both methods. Effect size estimate bias was reduced in MI methods by always including group membership even if this was unrelated to missingness. Surprisingly, under MAR and MCAR conditions in the assessed scenarios, MI offered no statistical advantage over CC methods.

**Conclusion:**

While MI must inherently accompany CC methods for intention-to-treat analyses, these findings endorse CC methods for per protocol risk difference analyses in these conditions. These findings provide an argument for the use of the CC approach to always complement MI analyses, with the usual caveat that the validity of the mechanism for missingness be thoroughly discussed. More importantly, researchers should strive to collect as much data as possible.

## Background

The randomized controlled trial (RCT) is considered the gold standard study design for evaluating the efficacy of a treatment or intervention in clinical and epidemiological research [1]. A well-designed and conducted RCT provides an efficient and unbiased estimate of effect size when all observations required by the study protocol have been obtained [1, 2], but difficulties can arise if some observations are missing. Missing outcome observations can create a specific and often considerable challenge for the statistical analysis. Incorrectly handled, they can result in biased and inefficient estimates of effect size, threatening the intrinsic strength of the RCT design and compromising the ability to draw valid inferences from the study findings [3].

Missing observations are least likely to occur at the baseline assessment, as many of the observations collected at this time are required not merely to provide a reference against which to measure efficacy but also to ensure that recruited participants meet the RCT inclusion/exclusion criteria. Missing observations tend to occur more frequently at follow-up assessments, when it is not uncommon for the primary outcome measure to be missing for some participants [4]. A considerable number of statistical methods have been, and continue to be, proposed for handling missing observations, but as yet universally accepted robust methods for handling missing data in RCTs do not exist [5].

Widely used analysis strategies include methods based on multiple imputation (MI), inverse probability weighting (IPW), doubly robust inverse probability weighting (DR-IPW) and maximum likelihood estimation (MLE). Despite the considerable body of literature on such methods, many researchers continue to use the simplest and most expedient approach of simply excluding from the statistical analyses all participants for whom the outcome measure is missing. This analytical method, commonly referred to as complete case (CC) analysis, is the default approach in many statistical packages [2, 6, 7]. There is, however, well-documented evidence that a CC analysis may yield biased and inefficient estimates of effect size, especially when the missing data levels are high, irrespective of the type and/or pattern of missingness [2, 8, 9].

The exact impact of a CC analysis on effect size estimates in different situations is poorly understood and has not been explored in detail [10]. This is a key gap in our knowledge. The choice of the most appropriate analytical method for handling missing outcome data in any RCT is ideally informed by the mathematical properties of the different analysis methods available, the missing observation pattern present and an understanding of the mechanism(s) that led to the missing observations [11]. Furthermore, it cannot be assumed that increased methodological complexity leads to less bias; there are known situations in which MI methods produce identical bias levels to those of a CC analysis [7, 12].

Many RCTs use a binary outcome measure (survived/died, outcome absent/present, treatment failure/success), in which case effect size is estimated using an odds ratio (OR), risk ratio (RR) or risk difference (RD) [13]. The RD is becoming increasingly popular due to its ease of interpretation. Several simulation studies have compared methods for handling missing binary outcome observations when effect size is estimated using an OR [2, 12, 14], but we are not aware of any publications on how missing observation methods perform when effect size is estimated using an RD. As OR and RD modelling use different mathematical algorithms, the results from an OR model cannot necessarily be extrapolated to an RD model.

In this paper, we use simulation methods to compare the performance of CC and MI to estimate effect size using the RD in RCTs with missing binary outcome observations and explore which method is preferable for various missing observation patterns and effect size levels.

## Methods

Simulated data sets were generated to compare the impact of CC and MI analytical approaches on effect size estimation in a two-group RCT with a binary outcome measure when some outcome observations are missing, across a range of effect sizes and missing outcome levels as detailed below. The parameters used in each simulated data set were based on the results of a malaria efficacy RCT conducted in Malawi between 2003 and 2006 [15].

For each effect size and missing outcome level combination examined, 5000 data sets were simulated. To reflect the parent malaria RCT, the sample size for each data set was 200 participants, with 100 subjects randomized to the intervention and the control groups respectively. The binary outcome was generated using a logit model to achieve a range of effect sizes (treatment group differences); a random process was then used to delete a prespecified proportion of outcomes.

For each participant in both treatment groups, baseline values were generated to represent their age, weight (wt), hemoglobin (hb) level and malaria parasitaemia count (para) using a multivariate normal distribution. Haemoglobin level was generated untransformed, but as body weight, age and parasitaemia counts had skewed distributions in the parent RCT, these variables were generated using a logarithmic scale, with their parameter values (means, variances and covariance) estimated from the log-transformed variable values in the parent RCT [16]. These variables are generally expected to be related to the outcome (adequate clinical and parasitological response). The matrices of parameters used to simulate the baseline covariate observations were:

$$ \mathbf{X}=\left[\begin{array}{l}{ \log}_e(Age)\\ {}hb\\ {} \log (Weight)\\ {} \log (Parasitaemia)\end{array}\right],\boldsymbol{\upmu} =\left(\begin{array}{c}\hfill 3.15\hfill \\ {}\hfill 9.32\hfill \\ {}\hfill 2.40\hfill \\ {}\hfill 10.70\hfill \end{array}\right)\ \boldsymbol{\upsigma} =\left(\begin{array}{c}\hfill 0.42\hfill \\ {}\hfill 1.66\hfill \\ {}\hfill 0.18\hfill \\ {}\hfill 1.50\hfill \end{array}\right)\ \mathrm{and}\ \boldsymbol{\uprho} =\left(\begin{array}{cccc}\hfill 1.00\hfill & \hfill 0.09\hfill & \hfill 0.16\hfill & \hfill 0.02\hfill \\ {}\hfill 0.09\hfill & \hfill 1.00\hfill & \hfill 0.40\hfill & \hfill 0.20\hfill \\ {}\hfill 0.16\hfill & \hfill 0.40\hfill & \hfill 1.00\hfill & \hfill 0.05\hfill \\ {}\hfill 0.02\hfill & \hfill 0.20\hfill & \hfill 0.05\hfill & \hfill 1.00\hfill \end{array}\right) $$

where:

***X*** is a vector of the four covariates log(age), haemoglobin (hb), log(body weight) and log(parasitaemia);

***μ*** and ***σ*** are vectors of the mean and standard deviation values respectively for each of log(age), haemoglobin, log(body weight) and log(parasitaemia);

***ρ*** is a matrix of the correlations between each pair combination of the baseline covariates.

To maintain the skewness of these covariates found in the parent RCT, the lognormal generated variables were transformed (exponentiated) back into their original form prior to analysis. The model estimated the (binary) outcome as a function of treatment group, age and haemoglobin.

The binary outcome was then simulated for each of the two groups to achieve the desired efficacy (treatment success) rates using a Bernoulli (π_*i*_) distribution, where π_*i*_ is the mean proportion of subjects with treatment success (efficacy) in group *i*, for *i* = A, B. This resulted in simulated binary outcome data with π_*i*_ success rate (efficacy) in group *i*.

The efficacies of treatments A and B respectively were generated using Bernoulli distributions as follows (Y = 1 denotes treatment success and T = treatment):

*for response rates of 85 % in treatment A versus 60 % in treatment B*

$$ \begin{array}{l}\mathrm{Y}=\mathrm{B}\mathrm{ernoulli}\left[ Pr\left(\mathrm{Y}=1\;\Big|\;\mathrm{T}=\mathrm{A}\right)=0.85\right]\\ {}=\mathrm{B}\mathrm{ernoulli}\left[ Pr\left(\mathrm{Y}=1\;\Big|\;\mathrm{T}=\mathrm{B}\right)=0.60\right]\end{array} $$

*for response rates of 98 % in treatment A versus 95 % in treatment B*

$$ \begin{array}{l}\mathrm{Y}=\mathrm{B}\mathrm{ernoulli}\left[ Pr\left(\mathrm{Y}=1\;\Big|\;\mathrm{T}=\mathrm{A}\right)=0.98\right]\\ {}=\mathrm{B}\mathrm{ernoulli}\left[ Pr\left(\mathrm{Y}=1\Big|\mathrm{T}=\mathrm{B}\right)=0.95\right]\end{array} $$

Four different imputation models were considered:

- model 1: log(weight), haemoglobin, log(age) and log(parasitaemia) were used to simulate the missing outcome observations.
- model 2: log(weight) was excluded leaving just haemoglobin, log(age) and log(parasitaemia).
- model 3: group membership was added to the covariates used in model 2.
- model 4: all of log(age), haemoglobin, log(age), log(parasitaemia) and group membership were used.

Imputations were conducted using the chained equations procedure [17]. Both 10 and 50 imputations were used in each multiple imputation procedure to provide information on the potential impact of increasing imputation rate.

All simulations and statistical summaries were performed using the Stata for windows software (version SE/11; StataCorp, College Station, TX, USA).

### Choice of effect size settings

Two different effect size settings were simulated.

1. 85 % for the treatment of interest (group A) and 60 % for the control treatment (group B).
  This scenario is not as unrealistic as it might appear. Relatively large effect sizes of this or a greater magnitude are not uncommon in malaria RCTs (see e.g. Bell et al. (2008) [15]). Furthermore, resistance is often underestimated when designing such trials, so sample size calculations are based on smaller differences than are actually observed. Consequently, sample sizes can be over-estimated, producing statistically significant findings even if the (binary) outcome is missing for as much as 30 % of participants. This setting was selected primarily, however, to avoid the model convergence problems that can occur when either group returns an effect rate close to the boundary (either 0 % or 100 %).
2. 98 % for the treatment of interest (group A) and 95 % for the control treatment (group B).

For this second setting, both effect rates were deliberately set close to the boundary value of 100 %, as this is a common situation in malaria treatment trials comparing highly efficacious artemisinin-based combination therapies.

### Choice of missing outcome settings

Consider an RCT with two treatment arms in which the primary outcome Y is a binary variable measured once, at the end of a fixed period of time of follow-up, for each patient. Let X denote the complete (uni- or multi-dimensional) covariate matrix, and let D be an indicator variable such that D = 1 if Y is missing and D = 0 if Y is observed.

Within this context, the following three missing data mechanisms defined by Rubin [18] were considered.

### Outcome missing at random (MAR)

An outcome observation was defined as MAR if the probability (Pr) of it being missing was dependent on the observed covariates X but independent of the specific value that theoretically should have been observed for that missing observation [18]. This is expressed mathematically as follows:

$$ Pr\left(\mathrm{D}=1\;\Big|\;\mathrm{Y},\;\mathrm{X}\right)= Pr\left(\mathrm{D}=1\;\Big|\;\mathrm{X}\right) $$

### Outcome missing completely at random (MCAR)

An outcome observation was defined as MCAR if the probability of it being missing was independent of both the observed covariates X and the specific value that theoretically should have been observed for that missing observation [18]. This is expressed mathematically as follows:

$$ Pr\left(\mathrm{D}=1\;\Big|\;\mathrm{Y},\;\mathrm{X}\right)= Pr\left(\mathrm{D}=1\right) $$

### Outcome missing not at random (MNAR)

An outcome observation was defined as MNAR if the probability of it being missing was dependent on the observed covariates X, the observed outcome values and the unobserved outcome values [18]. This is expressed mathematically as follows:

$$ Pr\left(\mathrm{D}=1\;\Big|\;\mathrm{Y},\;\mathrm{X}\right)= Pr\left(\mathrm{D}=1\;\Big|\;{\mathrm{Y}}^{\mathrm{obs}},\;{\mathrm{Y}}^{\mathrm{mis}},\;\mathrm{X}\right) $$

where Y^obs^ and Y^mis^ are the observed and missing outcome values respectively.

### Method used to simulate MAR, MCAR and MNAR scenarios

Three missing level settings were considered for each scenario: 5 %, 15 % and 30 %.

To generate binary outcome data that were MCAR, a random number with a uniform [0,1] distribution was generated for each participant in the simulated data set. The *p* % of participants with the smallest random numbers were then coded as having their outcome observation missing, *p* taking the values 5 %, 15 % or 30 % as appropriate.

The following logistic regression models were used to generate missing outcomes with MAR levels of 5 %, 15 % and 30 % respectively and which were dependent on group and weight:

$$ \begin{array}{l}\mathrm{logit}\left(\pi \right)=\left(0.872*\mathrm{treatment}\right)+\left(0.099*\mathrm{weight}\right)-4.666\\ {}\mathrm{logit}\left(\pi \right)=\left(0.299*\mathrm{treatment}\right)+\left(0.043*\mathrm{weight}\right)-2.409\\ {}\mathrm{logit}\left(\pi \right)=\left(0.148*\mathrm{treatment}\right)+\left(0.022*\mathrm{weight}\right)\;\hbox{--}\;1.18\end{array} $$

where π is the probability of an outcome being missing.

The models used to generate missing outcomes with MNAR levels of 5 %, 15 % and 30 % respectively were:

$$ \mathrm{logit}\left(\pi \right)=2.99*\mathrm{outcome};\mathrm{logit}\left(\pi \right)=1.89*\mathrm{outcome};\mathrm{logit}\left(\pi \right)=1.20*\mathrm{outcome} $$

The MAR and MNAR missing outcome indicators were thus generated with distributions:

$$ \begin{array}{l}\mathrm{Bernoulli}\;\left[1\;/\;\Big(1+ exp\right[1\left]\Big)\right]\;\mathrm{f}\mathrm{o}\mathrm{r}\;\mathrm{M}\mathrm{A}\mathrm{R}\\ {}\mathrm{Bernoulli}\;\left[1/\left(1+ exp\left\{-\left({\mathrm{b}}_3*\mathrm{outcome}\right)\right\}\right)\right]\;\mathrm{f}\mathrm{o}\mathrm{r}\;\mathrm{M}\mathrm{NAR}\end{array} $$

where b_1_, b_2_ and b_3_ are (regression) coefficients outlined in the missing outcome data logit models above and π is the probability of an outcome being missing.

For MNAR, the models resulted in participants with a successful (positive) outcome being more likely to have their outcome missing, creating a greater proportion of missing outcomes in the high efficacy group than in the group with low efficacy, in turn resulting in differential proportions of missing outcomes between the two study groups. This is realistic in the context of malaria trials, as successfully treated participants may have less incentive to return for their final assessment, particularly if doing so would be costly or time-consuming.

To minimize the problems of model non-convergence when generated effect sizes are close to the boundary, Cheung’s modified least squares method [19] was used to estimate risk differences (RDs). This method, which uses ordinary least squares (OLS) estimation together with Huber-White (H-W) robust standard errors, is suitable if interest is confined to the estimation of RDs but is not suitable theoretically if there is interest in predicting probabilities for individual patients, as estimated values can fall outside the probability range 0 to 1.

### Data model and model assessment criteria

The outcome of interest was modelled as a function of age, hb and group using the following logistic regression model:

$$ \mathrm{logit}\left[\mathrm{P}\left(\mathrm{Y}=1\Big|\mathrm{T}=\mathrm{t},\;\mathrm{h}\mathrm{b},\;\mathrm{age}\right)\right]={\mathrm{b}}_0+{\mathrm{b}}_1\;*\mathrm{T}+{\mathrm{b}}_2\;*\mathrm{h}\mathrm{b}+{\mathrm{b}}_3\;*\mathrm{age}, $$

where:

t = A or B;

b_0_, b_1_, b_2_, b_3_ are estimates of intercept, treatment effect, hb effect and age effect respectively.

Data model specification was identical for all scenarios; only the methods of handling cases that had missing outcomes were varied. For complete case analysis, all cases with missing outcomes were excluded from the statistical analyses.

The performance of the data models from the different approaches of missing data at each level of missing data were compared against three criteria: bias, statistical coverage and root-mean-squared error (RMSE).

## Results

Consistent with the findings of Schafer and Rubin [20, 21], the results obtained using 10 and 50 imputations were virtually identical, so only the results using 50 imputations are presented.

### Missing outcome observations MAR, MCAR and MNAR: 60 % versus 85 % efficacy

**Fig. 1:**
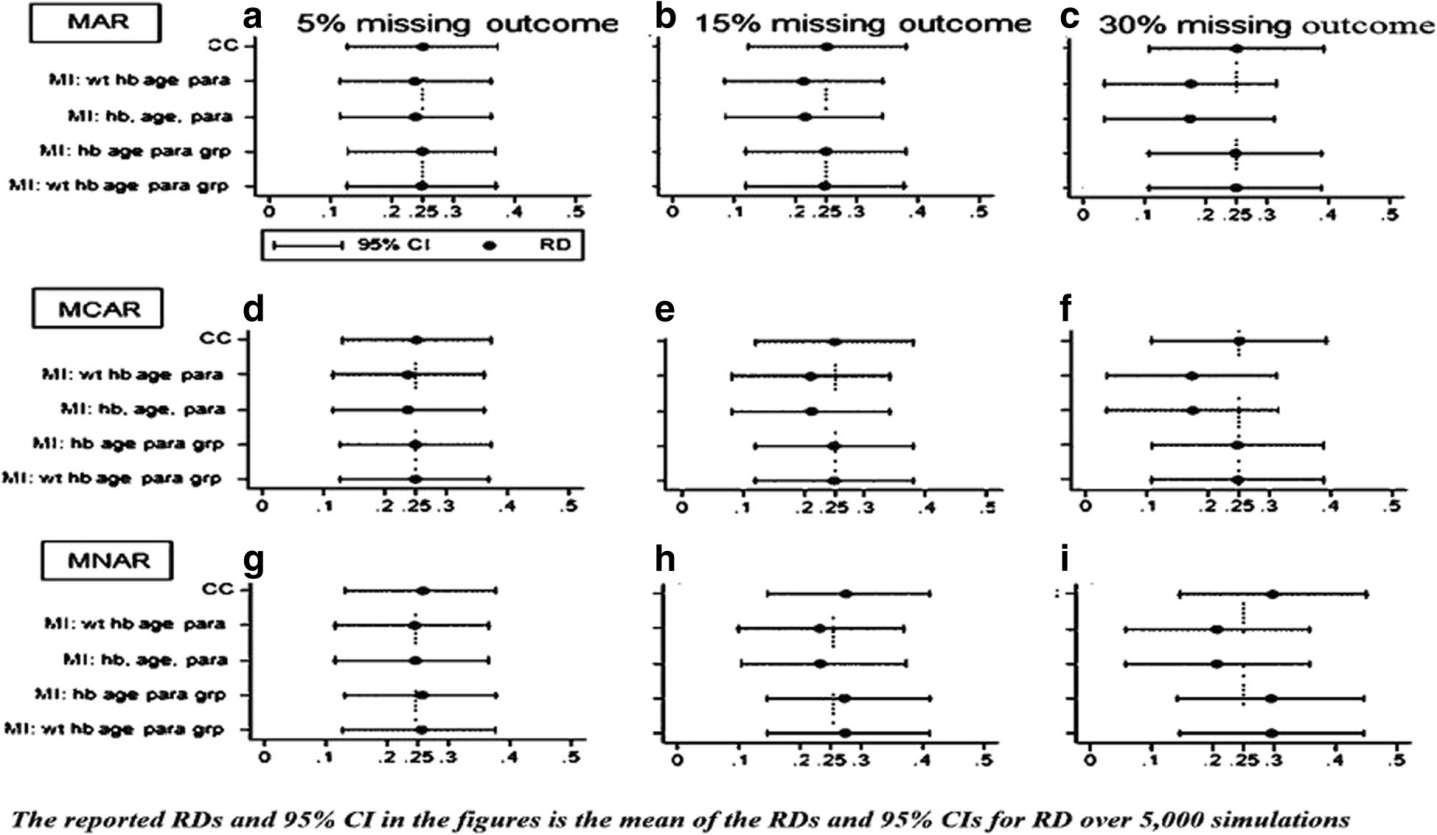
Estimated efficacy risk differences (60 % versus 85 %) MAR, MCAR, MNAR

When the missing outcome setting was MAR, effect size estimates became increasingly inefficient as the proportion of missing outcome observations increased. The RMSE values observed indicated that inefficiency levels were identical for both CC and MI methods. See Fig. 1.

Using CC methods, effect size estimates were unbiased for all assessed missing value levels. Using MI methods, when group membership was included in the imputation model, only a small degree of bias was observed in the effect size estimates. When group membership was excluded from the model, however, estimates were consistently negatively biased (i.e. effect size was consistently under-estimated), and the degree of bias increased as the proportion of missing outcome values increased.

Coverage was generally high for all models when missing value levels were small or moderate, but when the proportion of missing outcomes was extended to 30 %, coverage for the mis-specified MI models fell to around 88 % (detailed in Appendices 1, 2 and 3). In this context, imputation models not containing both of the variables weight and group membership were technically mis-specified, as it was these two variables that determined missingness. MI models containing both weight and group performed well for all missing outcome configurations, providing estimates that were only fractionally biased with good coverage of around 95 %, as did MI models that included group but excluded weight. MI models that included weight but excluded group, however, performed as badly as those MI models that included neither weight nor group.

With MCAR, the pattern of results was very similar to that for MAR. Coverage was generally high, remaining close to 95 % for all models at all missing value levels, except when the proportion of missing outcomes reached 30 %. At this point, as for MAR, coverage fell to around 88 % for mis-specified MI models, and increased levels of (negative) bias were observed.

Under the MNAR condition, RD estimates contained some degree of (usually but not exclusively positive) bias with both CC and MI methods, the degree of bias rising as the proportion of missing outcome observations increased. Coverage levels tended to be good, but with some deterioration at high missingness levels.

Detailed results for this scenario are provided in Appendices 1, 2 and 3 for MAR, MCAR and MNAR respectively.

### Missing outcome observations MAR, MCAR and MNAR: 95 % versus 98 % efficacy

**Fig. 2:**
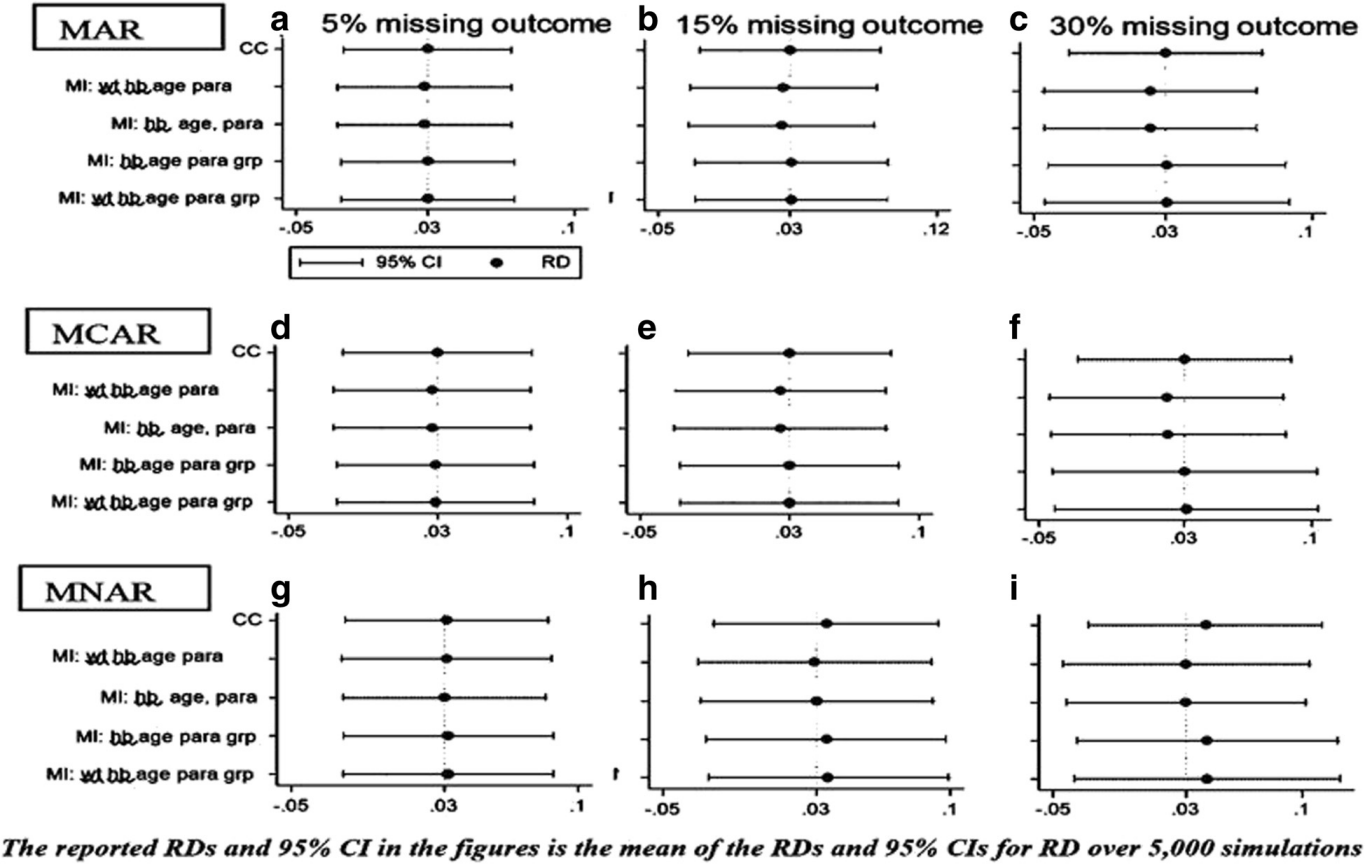
Estimated efficacy risk differences (95 % versus 98 %) MAR, MCAR, MNAR

See Fig. 2. When both efficacy levels were close to the 100 % boundary, coverage was poorest when there was no missing data (0.939 compared to the set nominal level of 0.950). All complete case (CC) analyses converged (Appendix 4, 5 and 6), but a small proportion of imputed analyses failed to converge or produce output in Stata. Non-convergence occurred more frequently with increasing proportions of missing outcome values (Appendix 4).

With these efficacy levels, all CC analyses converged while a small number of MI analyses failed to converge for all three missing data mechanisms (MAR, MCAR and MNAR). Non-convergence in MI analyses occurred more frequently with increasing proportions of missing outcome values. As the proportion of missing data increased, the standard errors of the effect size estimates increased and the efficiency of all analyses decreased in both CC and MI analyses, though this was less marked with the CC analyses.

The estimates of effect size were unbiased for all missing value levels using CC methods, and only small levels of bias were detected for those imputation models that included group membership for both MAR and MCAR missing data scenarios. For the MAR and MCAR missing data mechanisms, the bias in the effect size estimates was markedly greater using imputation models that did not incorporate group membership.

In the MNAR missing data mechanisms, for the 5 % missing level, all the models were generally unbiased with generally good coverage of around 95 %, but as missing levels increased, all models led to invalid inference of treatment effect. The level of bias increased with increasing missing levels for the CC models and the MI models, even for those that included group membership. The mis-specified models had unbiased estimates but provided invalid inference because the coverage was conservatively too high (close to 100 %) instead of the nominal 95 %.

Detailed results for this scenario are provided in Appendices 4, 5 and 6 for MAR, MCAR and MNAR respectively.

## Discussion

When binary outcome observations are missing that can be assumed to be MAR or MCAR, CC analysis methods were found to perform as well as, and often better than, MI methods, consistently producing unbiased RD estimates. This finding is consistent with those reported by Groenwold [14], who examined missing binary outcomes in an RCT setting using odds ratio as the effect size estimate of interest. The efficient estimates obtained from CC compared to MI are a counter-intuitive and surprising finding.

The loss of statistical efficiency in CC analyses is attributed directly to the reduction in the effective sample size that occurs with this method, as has been reported previously [8]. A surprising and unexpected finding, however, was that the loss in efficiency using CC analysis methods was consistently no worse than, and often better than, that observed using MI methods. Theoretically, MI methods are expected to yield unbiased standard errors, because sample size is maintained and the uncertainty in the imputed values is fully accounted for [8].

A plausible explanation for this unexpected efficiency finding is that the MI procedures also increase the variability in the outcome values that inflates the standard error of the effect size estimate. This increase in variability is likely caused by the random component that is added to missing outcome values during the imputation process.

No convergence problems were experienced using CC analyses when missing binary outcomes could be assumed to be MAR or MCAR, although some problems were experienced when missingness was MNAR. In contrast, convergence problems occurred under both the MAR and MCAR conditions when imputation models were used, particularly when both efficacy rates were close to the parameter boundaries. This was caused by all imputed values being occasionally allocated to the same outcome value across all imputations when efficacy levels in both groups are close to the boundary, which results in zero standard errors for the effect size estimate, a phenomenon referred to as ’perfect prediction’ [17]. Perfect prediction can arise in any generalized linear model that has a categorical outcome [22]. The usual reason for perfect prediction in an MI analysis is that all imputed values take the same value for all participants across the imputations, resulting in zero between-imputation variance. In this situation the calculation of degrees of freedom would involve division by zero, which may result in non-convergence. White et al. have suggested that perfect prediction problems can be a result of the flat likelihood [22].

This problem of perfect prediction can be drastically reduced in Stata by using the command option ’augment’, which causes an augmented regression to be performed [22].

Another striking finding under the MAR and MCAR conditions was that the inclusion of treatment group membership in the imputation process played a crucial role in improving its performance. Excluding this variable from the imputation process produced biased estimates of the adjusted efficacy RD. If missingness is related to some covariates, the absence of those covariates in the imputation model appeared to have little impact on bias levels for the effect size estimate, provided group was included in the imputation calculations. Less predictably, including treatment group membership would also appear to be paramount over all other factors, even when this is not related to missingness.

Under the MNAR condition, when missingness is related to treatment group membership and outcome, both CC and MI analyses produce biased estimates of effect size. Furthermore, the inclusion of group in a multiple imputation analysis will tend to lead to positive bias away from the null hypothesis. Thus, MNAR binary outcomes appear generally to over-estimate effect size, with the possible exception of mis-specified MI models, a counter-intuitive finding that requires further research. The results from the two mis-specified imputation models are presented in this paper to emphasize that, when assuming MI, care must be taken when selecting the imputation model, as using a poor imputation model can bias the effect size estimates.

This study has demonstrated that in the presence of missing binary outcome observations in an RCT with a single follow-up endpoint of interest, CC and MI analysis methods performed very similarly under the three missingness assumptions examined, except when an inappropriate imputation model was adopted, in which case the MI RD estimates obtained were generally inferior to those generated by a CC analysis. These findings indicate that MI methods offered no advantages over the much easier to apply CC method in the scenarios considered.

There are, however, other factors to be considered when analysing the findings of an RCT. The intention-to-treat principle (ITT) is now the standard procedure for the primary evaluation of an RCT. Under this principle, the use of MI methods may be preferable on the grounds that they retain all patients in the statistical evaluation, whereas the CC methods exclude all patients for whom the outcome measure could not be recorded.

A reasonable compromise might be to perform an MI analysis as the primary ITT analysis, following a rigorous exploration of the likely underlying reasons for the missingness in the outcome measure. Inappropriate imputation models can lead to RD estimates that are inferior to those from a CC analysis, so a ’non-parsimonious’ approach to this MI analysis is essential (i.e. as many covariates as possible must be included in the imputation process). In addition, group membership must be included in the imputation model; otherwise, there is an increased risk of bias, even when missingness is not in fact related to group membership. A secondary CC analysis could then be performed as part of the per protocol analyses.

MI methods have no place in a per protocol risk difference analysis. CC methods yield unbiased effect size estimates and are less prone to the problem of perfect prediction when effect sizes stray close to a boundary. MI methods are more suitable when the missingness is MNAR and thus have an important role both in sensitivity analyses and when the outcome of interest is collected at several points during a study. Unfortunately, the importance of sensitivity analyses is frequently under-valued [23].

From a reporting perspective, the general research community is likely to be skeptical of a statistical evaluation of an RCT that presents only a CC analysis in which 15–30 % of outcome measures are missing. The findings would most likely be perceived as potentially biased even when the mechanism of missingness is clearly MCAR, as in sample processing errors, a point that has been highlighted in these simulations.

Of course, for an RCT with efficacy levels away from the boundary, participants for whom the (binary) outcome measure is missing contribute nothing more than a collection of baseline characteristics; imputing the missing outcomes does not provide any empirical information about the relation between the exposure and the outcome.

While these findings appear to strengthen the argument in favour of CC analyses, as has been suggested by Liublinska and Rubin [24], they do not prove that CC methods are necessarily appropriate in all situations. This paper considers just the case of an RCT with a single binary outcome measurement and RD estimation (a common practical scenario in malaria studies of efficacy); in more complex study designs (for example, longitudinal or cluster randomized studies), and particularly when there are missing observations across many variables, MI methods remain superior to CC methodologies.

## Conclusions

MI analyses must be the primary analyses for the intention-to-treat analyses. These findings provide an argument for the use of the CC approach to always complement MI analyses, with the usual caveat that the validity of the mechanism for missingness be thoroughly discussed. The study also endorses CC methods for per protocol risk difference analyses under these conditions. Pragmatically, while the evidence favours the adoption of a CC analysis when (binary) outcome measures are missing, the reality is that the compromise approach suggested above of a carefully considered primary MI analysis followed by a secondary (essentially sensitivity) CC analysis may be most sensible in terms of getting the findings of such a RCT accepted, even in those situations in which the missing outcomes are ’clearly’ MCAR or MAR.

More importantly, researchers should strive to collect as much data as possible.

### Limitations

Different coefficients were used for the treatment group and weight variables to generate different percentages of missing data. Some confounding is thus possible between the impact of including/not including both group membership and weight in the MI analyses and the effect of increasing the percentage of missing data. A better approach might have been to fix the effects of treatment group and weight on the missing outcome and then only to allow β_0_ to vary to achieve different percentages of missing data. This was an oversight at the study design stage, and we are grateful to a reviewer for pointing out this potential interpretation issue. In addition, the effect size used for weight was small compared to that used for treatment group; it is possible that this may explain in part why including/excluding treatment effect from the imputation had a considerably greater impact than including/excluding weight.

We also acknowledge that the inflated RMSEs with MI analyses may well be due to the discrepancy between the imputation and analysis models. That fact that logistic regression was used to impute missing outcomes while the OLS regression was used to analyse binary outcome data might impact the size of the RMSEs in the MI analyses.

## Abbreviations

CC, complete case (analysis); DR-IPW, doubly robust inverse probability weighting; hb, haemoglobin; H-W, Huber-White; IPW, inverse probability weighting; ITT, intention-to-treat; MAR, missing at random; MCAR, missing completely at random; MI, multiple imputation; MLE, maximum likelihood estimation; MNAR, missing not at random; OLS, ordinary least squares; OR, odds ratio; para, parasitaemia; RCT, randomized controlled trial; RD, risk difference; RMSE, root-mean-squared error; RR, risk ratio; wt, weight

## Appendices

### Appendix 1

MAR and efficacy rate 85 % versus 60 % (RD 0.250). The estimated efficacy differences, coverage and bias for 5 %, 15 % and 30 % averages of 5000 simulated data sets with 50 imputations are shown.

**Table 1:** MAR and efficacy rate 85 % versus 60 % (RD 0.250): estimated efficacy differences, coverage and bias for 5 %, 15 % and 30 % averages of 5000 simulated data sets, 50 imputations

Model	RD (RMSE)	Coverage	Bias
*All outcomes recorded:*	0.250 (0.061)	0.950	0.000
*5 % of outcomes missing:*			
CC	0.250 (0.063)	0.946	0.000
MI: wt, hb, age, para	0.237 (0.063)	0.958	-0.013
MI: hb, age, para	0.238 (0.063)	0.961	-0.012
MI: hb, age, para, group	0.249 (0.062)	0.949	-0.001
MI: wt, hb, age, para, group	0.248 (0.062)	0.945	-0.002
*15 % of outcomes missing*			
CC	0.250 (0.066)	0.945	0.000
MI: wt, hb, age, para	0.212 (0.066)	0.944	-0.038
MI: hb, age, para	0.214 (0.066)	0.947	-0.036
MI: hb, age, para, group	0.249 (0.066)	0.948	-0.001
MI: wt, hb, age, para, group	0.247 (0.066)	0.949	0.003
*30 % of outcomes missing*			
CC	0.250 (0.073)	0.948	0.000
MI: wt, hb, age, para	0.175 (0.071)	0.887	-0.075
MI: hb, age, para	0.174 (0.071)	0.879	-0.076
MI: hb, age, para, group	0.248 (0.072)	0.946	-0.002
MI: wt, hb, age, para, group	0.249 (0.072)	0.946	-0.001

### Appendix 2

MCAR and efficacy rate 85 % versus 60 % (RD 0.250). The estimated efficacy differences, coverage and bias for 5 %, 15 % and 30 % averages of 5000 simulated data sets with 50 imputations are shown.

**Table 2:** MCAR and efficacy rate 85 % versus 60 % (RD 0.250): estimated efficacy differences, coverage and bias for 5 %, 15 % and 30 % averages of 5000 simulated data sets, 50 imputations

Model	RD (RMSE)	Coverage	Bias
*All outcomes recorded:*	0.250 (0.061)	0.950	0.000
*5 % of outcomes missing*			
CC	0.250 (0.062)	0.946	0.000
MI: wt, hb, age, para	0.237 (0.063)	0.957	-0.013
MI: hb, age, para	0.237 (0.063)	0.957	-0.013
MI: hb, age, para, group	0.249 (0.063)	0.950	-0.001
MI: wt, hb, age, para, group	0.249 (0.062)	0.944	-0.001
*15 % of outcomes missing*			
CC	0.250 (0.066)	0.946	0.000
MI: wt, hb, age, para	0.211 (0.066)	0.946	-0.039
MI: hb, age, para	0.212 (0.066)	0.944	-0.038
MI: hb, age, para, group	0.249 (0.066)	0.950	-0.001
MI: wt, hb, age, para, group	0.249 (0.066)	0.941	-0.001
*30 % of outcomes missing*			
CC	0.250 (0.073)	0.946	0.000
MI: wt, hb, age, para	0.173 (0.071)	0.880	-0.077
MI: hb, age, para	0.174 (0.071)	0.891	-0.076
MI: hb, age, para, group	0.247 (0.072)	0.944	-0.003
MI: wt, hb, age, para, group	0.248 (0.072)	0.948	-0.002

### Appendix 3

MNAR and efficacy rate 85 % versus 60 % (RD 0.250). The estimated efficacy differences, coverage and bias for 5 %, 15 % and 30 % averages of 5000 simulated data sets with 50 imputations are shown.

**Table 3:** MNAR and efficacy rate 85 % versus 60 % (RD 0.250): estimated efficacy differences, coverage and bias for 5 %, 15 % and 30 % averages of 5000 simulated data sets, 50 imputations

Model	RD (RMSE)	Coverage	Bias
*All outcomes recorded:*	0.250 (0.061)	0.950	0.000
*5 % of outcomes missing*			
CC	0.258 (0.063)	0.942	+0.008
MI: wt, hb, age, para	0.244 (0.064)	0.962	-0.006
MI: hb, age, para	0.245 (0.064)	0.958	-0.005
MI: hb, age, para, group	0.257 (0.063)	0.946	+0.007
MI: wt, hb, age, para, group	0.256 (0.063)	0.946	+0.006
*15 % of outcomes missing*			
CC	0.274 (0.068)	0.932	+0.024
MI: wt, hb, age, para	0.231 (0.069)	0.975	-0.019
MI: hb, age, para	0.232 (0.069)	0.975	-0.018
MI: hb, age, para, group	0.272 (0.068)	0.931	+0.028
MI: wt, hb, age, para, group	0.273 (0.068)	0.928	+0.023
*30 % of outcomes missing*			
CC	0.298 (0.078)	0.895	+0.048
MI: wt, hb, age, para	0.206 (0.077)	0.974	-0.044
MI: hb, age, para	0.206 (0.077)	0.974	-0.044
MI: hb, age, para, group	0.295 (0.078)	0.901	+0.045
MI: wt, hb, age, para, group	0.296 (0.077)	0.898	+0.046

### Appendix 4

MAR and efficacy rate 98 % versus 95 % (RD 0.030). The estimated efficacy differences, coverage and bias for 5 %, 15 % and 30 % averages of number of simulated data sets that converged of the 5000 data sets, with 50 imputations, are shown.

**Table 4:** MAR and efficacy rate 98 % versus 95 % (RD 0.030): estimated efficacy differences, coverage and bias for 5 %, 15 % and 30 % averages of number of simulated data sets that converged of the 5000 data sets, 50 imputations

Model	No. of data sets*	RD (RMSE)	Coverage	Bias
*All outcomes recorded:*	5,000	0.030 (0.026)	0.939	0.000
*5 % of outcomes missing*				
CC	5,000	0.030 (0.026)	0.940	0.000
MI: wt, hb, age, para	4,982	0.027 (0.027)	0.951	-0.003
MI: hb, age, para	4,988	0.027 (0.027)	0.955	-0.003
MI: hb, age, para, group	4,989	0.029 (0.027)	0.946	-0.001
MI: wt, hb, age, para, group	4,986	0.029 (0.027)	0.947	-0.001
*15 % of outcomes missing*				
CC	5000	0.030 (0.028)	0.942	0.000
MI: wt, hb, age, para	4969	0.025 (0.029)	0.970	-0.005
MI: hb, age, para	4983	0.025 (0.029)	0.963	-0.005
MI: hb, age, para, group	4981	0.030 (0.030)	0.965	0.000
MI: wt, hb, age, para, group	4982	0.030 (0.030)	0.965	0.000
*30 % of outcomes missing*				
CC	5,000	0.030 (0.030)	0.937	0.000
MI: wt, hb, age, para	4,893	0.020 (0.033)	0.970	-0.010
MI: hb, age, para	4,949	0.021 (0.033)	0.98	-0.009
MI: hb, age, para, group	4,945	0.030 (0.037)	0.967	0.000
MI: wt, hb, age, para, group	4,938	0.031 (0.037)	0.971	+0.001

*Number of data sets for which convergent analysis was achieved

### Appendix 5

MCAR and efficacy rate 98 % versus 95 % (RD 0.030). The estimated efficacy differences, coverage and bias for 5 %, 15 % and 30 % averages of number of simulated data sets that converged of 5000 data sets, with 50 imputations, are shown.

**Table 5:** MCAR and efficacy rate 98 % versus 95 % (RD 0.030): estimated efficacy differences, coverage and bias for 5 %, 15 % and 30 % averages of number of simulated data sets that converged of 5000 data sets, 50 imputations

Model	No. of data sets*	RD (RMSE)	Coverage	Bias
*All outcomes recorded:*	5,000	0.030 (0.026)	0.939	0.000
*5 % of outcomes missing*				
CC	5,000	0.030 (0.026)	0.940	0.000
MI: wt, hb, age, para	4,977	0.028 (0.027)	0.948	-0.002
MI: hb, age, para	4,982	0.028 (0.027)	0.957	-0.002
MI: hb, age, para, group	4,992	0.030 (0.027)	0.953	0.000
MI: wt, hb, age, para, group	4,988	0.030 (0.027)	0.951	0.000
*15 % of outcomes missing*				
CC	5,000	0.030 (0.028)	0.944	0.000
MI: wt, hb, age, para	4,962	0.026 (0.029)	0.970	-0.004
MI: hb, age, para	4,969	0.025 (0.029)	0.970	-0.005
MI: hb, age, para, group	4,984	0.031 (0.030)	0.957	+0.001
MI: wt, hb, age, para, group	4,972	0.031 (0.030)	0.961	+0.001
*30 % of outcomes missing*				
CC	5,000	0.030 (0.030)	0.940	0.000
MI: wt, hb, age, para	4,896	0.021 (0.033)	0.969	-0.009
MI: hb, age, para	4,937	0.021 (0.033)	0.978	-0.009
MI: hb, age, para, group	4,948	0.031 (0.037)	0.971	+0.001
MI: wt, hb, age, para, group	4933	0.031 (0.038)	0.967	+0.001

*Number of data sets for which convergent analysis was achieved

### Appendix 6

MNAR and efficacy rate 98 % versus 95 % (RD 0.030). The estimated efficacy differences, coverage and bias for 5 %, 15 % and 30 % averages of number of simulated data sets that converged of the 5000 data sets, with 50 imputations, are shown.

**Table 6:** MNAR and efficacy rate 98 % versus 95 % (RD 0.030): estimated efficacy differences, coverage and bias for 5 %, 15 % and 30 % averages of number of simulated data sets that converged of the 5000 data sets, 50 imputations

Model	No. of data sets*	RD (RMSE)	Coverage	Bias
*All outcomes recorded:*	5,000	0.030 (0.026)	0.939	0.000
*5 % of outcomes missing*				
CC	5,000	0.031 (0.027)	0.947	+0.001
MI: wt, hb, age, para	4,989	0.031 (0.028)	0.960	+0.001
MI: hb, age, para	4,988	0.030 (0.027)	0.961	0.000
MI: hb, age, para, group	4,992	0.032 (0.028)	0.950	+0.002
MI: wt, hb, age, para, group	4,992	0.032 (0.028)	0.955	+0.002
*15 % of outcomes missing*				
Complete Case	5,000	0.035 (0.030)	0.951	+0.005
MI: wt, hb, age, para	4,985	0.029 (0.031)	0.981	0.000
MI: hb, age, para	4,990	0.030 (0.031)	0.981	-0.001
MI: hb, age, para, group	4,991	0.035 (0.032)	0.960	+0.005
MI: wt, hb, age, para, group	4,989	0.036 (0.032)	0.961	+0.006
*30 % of outcomes missing*				
CC	5,000	0.042 (0.036)	0.950	+0.012
MI: wt, hb, age, para	4,982	0.030 (0.038)	0.992	0.000
MI: hb, age, para	4,991	0.030 (0.037)	0.991	0.000
MI: hb, age, para, group	4,988	0.043 (0.040)	0.965	+0.013
MI: wt, hb, age, para, group	4,991	0.043 (0.041)	0.965	+0.013

*Number of data sets for which convergent analysis was achieved
